# Ginkgo Biloba Extract in an Animal Model of Parkinson’s Disease: A Systematic Review

**DOI:** 10.2174/1570159X11311040006

**Published:** 2013-07

**Authors:** K. Tanaka, R. F. S.- Galduróz, L. T. B. Gobbi, J. C. F. Galduróz

**Affiliations:** 1 Department of Psychobiology, Universidade Federal de São Paulo, São Paulo Brazil;; 2 Department of Physical Education, Universidade Estadual Paulista, Rio Claro, Brazil;; 3 Center of Mathematics, Computing and Cognition, Universidade Federal do ABC, Santo André, Brazil

**Keywords:** EGb761, Ginkgo biloba extract, Parkinson’s disease, Parkinsonism, Systematic review.

## Abstract

Although the exact cause of neuronal loss in Parkinson’s disease is not known, evidence points to oxidative stress and the production of reactive oxygen species as the main events that occur in the substantia nigra pars compacta of the brain of parkinsonians. EGb761 is an extract of the leaves from the Ginkgo biloba tree that has been reported as an antioxidant and neuroprotective agent. The objective of this work was to perform a systematic review of the studies that analysed the effect of Ginkgo biloba extract on Parkinson’s disease or Parkinsonism. This research was conducted using the following databases: Medline, PsycInfo, Cinahl, Sigle, Lilacs, Scielo, Cochrane Library, and Embase. Initially, we selected 32 articles. After a more detailed analysis, only 10 articles remained. One of the hypotheses for the positive effect of EGb761 on Parkinson’s disease is the reduction or inhibition of monoamine-oxidase activity. This enzyme metabolises dopamine, inducing the formation of free radicals, which in turn damage nigrostriatal neurons. Another hypothesis is that the neuroprotective effect of EGb761 against 6-hydroxydopamine, 1-methyl-4-phenyl-1,2,3,6-tetrahydropyridine and MPP+ toxins. As there are few studies on the effect of EGb761 on humans, this review could contribute new data to further the discussion of this issue.

## INTRODUCTION

Parkinson’s disease (PD) is a neurodegenerative pathology characterised by the loss of dopaminergic neurons, mainly in the *substantia nigra pars compacta, *and the resulting depletion of the neurotransmitter, dopamine (DA), in the *striatum*, which leads to motor performance decrement [1,2]. Although the exact cause of neuron loss in PD is not known, evidence points to oxidative stress and the production of oxygen reactive species [3-5].

No effective treatment for PD exists, as therapeutic drugs do not stop the progression of the disease and some produce undesirable side effects. Therefore, it is crucial that researchers develop new neuroprotective agents that aim to reduce or prevent the progression of PD.

EGb761 is an extract from the leaves of a tree known as *Ginkgo biloba.* It has been reported to be an antioxidative and neuroprotective agent in a variety of conditions, such as ischemia [6], oxidative stress [7] and induced β-amiloid toxicity [8]. EGb761 contains 24% flavonoids and 6% terpenoids (known as ginkgolides and bilobalides, respectively) [9]. The molecular weight of EGb761 is relatively low, allowing it to pass through the hematoencephalic barrier; hence, EGb761 presents a wide range of pharmacological actions on the central nervous system [10]. 

Clinically, the effects of *Ginkgo biloba* extract have been tested in a variety of neurological disorders, including PD, dementia, vaso-occlusive and cochlea-vestibular disorders [11,12].

Therefore, the objective of the present research was to perform a systematic review of the studies that analysed the effect of *Ginkgo biloba* extract on Parkinson’s disease or Parkinsonism.

## MATERIALS AND METHODS

### Design

We conducted a systematic review that analysed the following databases: Medline, Cinahl, PsycInfo, Sigle, Lilacs, Scielo, Cochrane Library, and Embase. The key words and the Boolean operators used were: Ginkgo OR Ginkgo biloba OR EGb761 OR EGb AND Parkinson’s disease OR Parkinson OR Parkinsonism OR Parkinsonian OR 6-OHDA OR 6-hydroxydopamine OR MPTP OR MPP^+^ OR 1-methyl-4-phenyl-1,2,3,6-tetrahydropyridine.

The search was performed in October of 2012. 

### Inclusion and Exclusion Criteria 

We included randomised and controlled studies with animals and humans, as well as those that utilised EGb761 at any doses. Reviews of the literature and non-controlled studies were excluded. We included only English language papers.

## RESULTS 

**Fig. (1):**
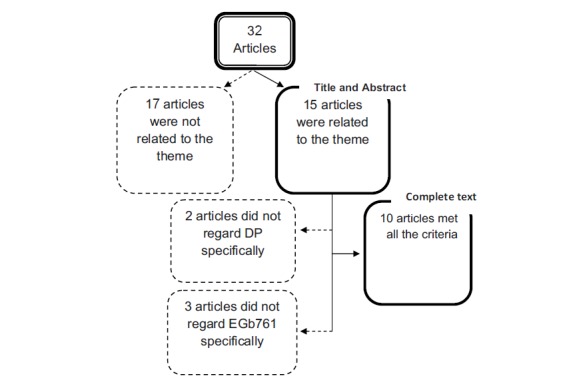
Stages of selection of the articles included in the systematic review.

According to the objective of this review, we initially selected 32 articles. After we performed an in-depth analysis based on the inclusion and exclusion criteria, only 10 articles remained (Fig. **1**). We found no studies in humans that fit the inclusion criteria.

Below, we provide a short description of the 10 studies selected for this review: 

Rojas *et al.* (2009) [12] analysed the protective effect of EGb761 against the MPP^+ ^neurotoxin, with the objective of relating it to copper in the brain. They administered EGb761 (10 mg/kg) to black C-57 mice, daily for 17 days. After this period, MPP^+^ was acutely administered (0.72 mg/kg), and copper was analysed by *graphite furnace atomic absorption spectrophotometry*. EGb761 protected the brain against the MPP^+^ neurotoxin. 

Another study by Rojas *et al.* (2008) [13] demonstrated the neuroprotective effect of EGb761 against MPTP-induced oxidative stress in C-57BL/6J mice. The mice received daily injections of vehicle (saline) or MPTP (30 mg/kg of body weight). EGb761 treatment was initiated 24 h after the last administration of MPTP. The concentrations of DA and tyrosine hydroxylase (TH) were analysed by immuno-histochemistry, and the authors quantified the number of TH-positive neurons, spontaneous locomotor activity, lipid peroxidation (LP), antioxidant enzymes, SOD, glutathione peroxidase (GPx) and glutathione reductase activity. These results suggest that EGb761 might attenuate the neuro-degeneration of the nigrostriatal pathway, causing an inhibitory effect on oxidative stress.

Ahmad *et al.* (2005) [14] evaluated the protective effect of EGb against the 6-OHDA toxin in rats. The animals were treated with EGb (50, 100, 150 mg/kg) for 3 weeks. On the 21^st^ day, 6-OHDA was injected in their *striatum*, while the control group received vehicle. The rotational behaviour, locomotor activity and muscular coordination of the rats were tested again three weeks after receiving 6-OHDA. Six weeks later, the researchers measured glutathione reductase (GSH). They concluded that EGb might be used as a therapeutic approach to prevent the neuronal loss resulting from Parkinsonism.

Kim *et al.* (2004) [15] analysed the neuroprotective effect of EGb761 against the 6-OHDA neurotoxin in the nigrostriatal dopaminergic pathway of rat brains. Pre-treatment consisted of daily administrations of EGb761 for one week. After this period, 6-OHDA was injected for one a week. The authors analysed the behaviour of the animals as well as brain dopamine levels using HPLC and histology. These results suggest that EGb761, through its neuroprotective effect, reduces the behavioural deficit caused by the 6-OHDA toxin in rats. 

Rojas *et al.* (2004) [16] analysed the effect of EGb761 on the striatal activity of the MAO as a neuroprotective mechanism against the MPP^+^. C-57 black mice were pre-treated with EGb761 (10 mg/kg), daily for 17 days, prior to receiving MPP^+^ (0.72 mg/kg). The MAO, DA striatal and TH activities of the mice were then analysed. These results suggest that supplementation with EGb761 might effectively reduce MAO activity, preventing MPP^+^ neurotoxicity.

Gagné *et al.* (2003) [17] investigated the role of oxidative stress in native neuronal and PC12 cells. They also measured 

flavonoids and β-estradiol (E_2_), as well as their capacity to reverse the damage caused by oxidation. The cells were pretreated with E_2_, kaempferol, quercetin, EGb761, or CP 202 for 24 h. After pretreatment, the cells were incubated with MPP^+^. These results show that both EGb761 and CP 202 were able to initiate recovery in the cells studied. 

Cao *et al.* (2003) [18] analysed whether the combination of levodopa and EGb could be a feasible strategy for the treatment of PD. Rats received an injection of 6-OHDA in the ventral tegmental area (VTA) and in the *substantia nigra* pars compacta (SNc). The authors studied the rotational behaviour, immunocytometry, and colour of Nissl bodies of the groups treated with levodopa and the combination of levodopa and EGb (100 mg/kg every day, E-D group). These results suggest that levodopa has a neurotoxic effect, and EGb reduces the toxicity induced by levodopa. Therefore, the combination of levodopa and EGb might be a better method to treat PD.

Yang *et al.* (2001) [19] observed the effects of EGb in MPTP-induced PD models (MPP^+^). Wistar rats were pretreated with EGb (50 or 100 mg/kg) for 19 days before, and one day after, the administration of MPTP. The authors analysed the amounts of malondialdehyde (MDA), SOD, and dopamine in the *substantia nigra* of rats. PC12 cell apoptosis was induced by MPP+, and the protective effect of EGb (25, 50, and 100 mg/L) was also observed. These results suggest that EGb has a protective effect in both *in vivo* and *in vitro* models of PD. The anti-oxidant and anti-apoptotic effects of EGb might be partially responsible for the neuroprotective effect of EGb.

Another study (Rojas *et al.*, 2001) [20] analysed the effects of pre-treatment with EGb761 on the effect of the MPP^+^ neurotoxin. C-57 black mice were pre-treated with EGb761 at different doses (0.63, 1.25, 2.5, 5 or 10 mg/kg) for 17 days. After pre-treatment, MPP^+^ (0.18, 0.36 or 0.72 mg/kg) was administered, and the mice were analysed 30 min, 1 h, 2 h and 24 h after MPP^+^ administration. Striatal DA content was analysed by HPLC at the highest doses of EGb761, at 2 h and 24 h after MPP^+^ administration. These results suggest that supplementation with EGb761 may effectively prevent oxidative stress. 

Wu and Zhu (1999) [21] observed the neuroprotective and neurorestorative effects of EGb761 and its two ginkgolide components, A (BN52020) and B (BN52021), on C-57BL/6J mice. The animals were treated with EGb761 (20, 50, 100 mg/kg), BN52020 or BN52021 (10 or 50 mg/kg), MPTP, or saline, and the extent of MAO-B inhibition was analysed. These results show that pretreatment with EGb761 can protect against MPTP. Moreover, the neuroprotective effect of EGb761 may be associated with its inhibitory effect on MAO in the brain. The characteristics and results of the studies reviewed are summarised in Table **1**.

## DISCUSSION

The results of this review show the beneficial effects of *Ginkgo biloba* extract in animal models of PD.

One potential mechanism to explain the positive effect of EGb761 on PD is the reduction or inhibition of MAO activity. This enzyme metabolises DA, inducing the formation of free radicals, which may cause damage to nigrostriatal neurons [22]. Rojas *et al.* (2004) [16] pre-treated mice with EGb761 and observed a preventive effect against MPP^+^, which increases the activity of the MAO. Wu and Zhu (1999) [21] observed similar effects. 

Another possibility regarding the neuroprotective effect of EGb761 is against the 6-OHDA, MPTP and MPP^+^ toxins. Specifically, the 6-OHDA is a toxin used in experimental models to determine dopaminergic function and assess the evolution of the activity of neuroactive drugs on the central dopaminergic system. Direct administration of 6-OHDA in the *substantia nigra* or *striatum* may cause degeneration of nigrostriatal neurons, which worsens motor function. The effects of this toxin are attributed to the formation of various oxidants and free radicals, lipid peroxidation, depletion of GSH and deficits in the mitochondrial complex [23]. Ahmad *et al.* (2005) [14] induced Parkinsonism using the 6-OHDA toxin after pre-treatment with EGb761, and they observed recovery of behavioural activity and cell integrity. Kim *et al.* (2004) [15] also conducted pretreatment with EGb761 before the introduction of 6-OHDA toxin. Their results suggest a neuroprotective effect, as the extract reduced behavioural deficits, such as akinesia, in rats. Cao *et al.* (2003) [18] compared the use of levodopa, with or without EGb761, in rats that were administered 6-OHDA *via *ingestion, and they observed that combined use reduces the toxicity of levodopa. 

MPP^+^ is an active metabolite of the MPTP toxin that induces parkinsonism by causing dopaminergic cell death through the inhibition of the mitochondrial complex I. Rojas *et al.* (2009) [12] treated mice with EGb761 before the injection of MPP^+^. Their results showed that the extract has a neuroprotective effect against this toxin, in addition to regulating the homeostasis of copper in the brain of the animal. In another study, Rojas *et al.* (2008) [13] used the MPTP toxin to induce Parkinsonism in mice, and the animals were treated with EGb761. Their results suggest that EGb761 attenuates neurodegeneration in the nigrostriatal pathway and inhibits oxidative stress. Gagné *et al.* (2003) [17] investigated the antagonistic effect of EGb761 on oxidative stress caused by MPTP and the neural growth factor in PC12 cells. Their results suggest that the extract was able to repair the cells. Yang *et al.* (2001) [19] injected MPTP in rats that were pre-treated with EGb761 to assess its neuroprotective effect. Their results showed a neuro-protective effect on PD models. Another study by Rojas *et al.* (2001) [20] also detected a neuroprotective effect EGb761 against MPP^+^, which was due to its ability to protect the *striatum* and prevent DA depletion. 

These reports led us to believe that the a possible cause of PD or Parkinsonism might be oxidative stress, which could be effectively prevented by EGb761, as observed in the results obtained in this systematic review of the beneficial effects of the extract in animal models of PD. Oxidative stress, as observed in these studies, is a result of the oxidation of MPTP into MPP^+^ by MAO-B. This ion is a neurotoxin, and it builds up in the mitochondria of the dopaminergic neurons, blocking the activity of the respiratory chain. This inhibition causes the reversible loss of ATP and stimulates the production of free radicals that lead to neuronal loss [24,25]. Studies with humans are warranted, because they could aid in symptom reduction and improve the quality of life of individuals with Parkinson’s disease. Therefore, we might conclude that Ginkgo biloba extract could be a coadjutant in the treatment of Parkinson’s disease, with beneficial and/or protective effects.

## Figures and Tables

**Table 1.** Characteristics of Studies that Analysed the Effects of Ginkgo Biloba Extract on Parkinson's Disease

Author	Sample	Intervention	Evaluation	Results
[12] Rojas *et al.* (2009)	Male C-57 black mice. Group I: saline solution + saline solution; Group II: EGb761 + saline solution: Group III: saline solution + MPP+; Group IV: EGb761 + MPP+. (EGb761 = 10 mg/kg, i.p.; MPP+ = 0.72 mg/kg, icv)	Mice from Groups I and II were used as controls. After pretreatment, groups III and IV were injected with MPP+.	The striatum, midbrain, hippocampus, frontal cortex and cerebellum were removed, and their copper content was measured.	The protective effect of EGb761 against MPP+ neurotoxicity may be due, in part, to the regulation of copper homeostasis in the brain.
[13] Rojas *et al.* (2008)	Male C-57 black mice. Group I: saline solution + saline solution; Group II: saline solution + EGb761; Group III: MPTP + saline solution; Group IV: MPTP + EGb761. (MPTP = 30 mg/kg, i.p.; saline = 30 mg/kg, i.p.; EGb761 = 10, 40, 80 or 120 mg/kg, i.p.)	The groups received saline, EGb761 or MPTP for 18 days, and the mice were killed 24 h after the last administration.	-DA concentration; -TH; -LP; - GPx; -Immunohistochemistry and stereological quantisation of TH-positive neurons; - Spontaneous locomotor activity; -Antioxidant enzymes; -SOD activity. -Glutathione reductase activity.	EGb761 attenuates neurodegeneration of the nigrostriatal pathway and inhibits oxidative stress.
[14] Ahmad *et al.* (2005)	Male Wistar rats. Group I: saline; Group II: saline + EGb (50 mg/kg, i.p.); Group III: saline + EGb (100 mg/kg, i.p.); Group IV: saline + EGb (150 mg/kg, i.p.); Group V: 6-OHDA; Group VI: 6-OHDA + EGb (50 mg/kg, i.p.); Group VII: 6-OHDA + EGb (100 mg/kg, i.p.); Group VIII: 6-OHDA + EGb (150 mg/kg, i.p.). (saline = 2 µL; 6-OHDA = 10 µg/2 µL in 0.1% in ascorbic acid-saline into right striatum)	The rats were treated with EGb for 3 weeks. On day 21, 6-OHDA was injected, while the other groups received the vehicle.	-Rotational behaviour; -Locomotor activity; -Muscular coordination; -TBARS; -GSH.	EGb restored the rotations and deficits in locomotor activity, muscular coordination, increased the generation of TBARS and depleted GSH content in the substantia nigra.
[15] Kim *et al.* (2004)	Male Sprague-Dawley rats. Group I: saline (0.9% NaCl) + 6-OHDA; Group II: EGb761 (50 mg/kg, i.p.) + 6-OHDA; Group III: EGb761 (100 mg/kg, i.p.) + 6-OHDA. (6-OHDA = 3.5 µL, injected striatum)	The rats were pretreated with EGb761 or vehicle, daily for a week, before 6-OHDA lesion. Treatment continued for a week after the lesion.	-Behavioural assessment; -Brain catecholamine determination by HPLC; -Histology.	EGb761 reduces behavioural deficits and is suggested as a treatment for PD.
[16] Rojas *et al.* (2004)	Male C-57 black mice. Group I: saline solution + saline solution; Group II: EGb761 + saline solution; Group III: saline solution + MPP+; Group IV: EGb761 + MPP+. (EGb761 = 10 mg/kg, i.p.; MPP+ = 0.72 mg/kg, icv)	The mice were pretreated with EGb761, daily for 17 days, followed by administration of MPP+.	-MAO activity; -Striatal DA; -TH activity.	EGb761 prevented the enhancement of striatal MAO activity, the striatal dopamine-depleting effect and the reduction in striatal tyrosine hydroxylase activity.
[17] Gagn&eacute; *et al.* (2003)	PC12 cells Group I: saline + MPP+ (5 m*M*); Group II: E2 (10-7 *M*) + MPP+(5 m*M*); Group III: Kaempferol (10-7 *M*) + MPP+(5 m*M*); Group IV: Quercetin (10-7 *M*) + MPP+(5 m*M*); Group V: Cp 202 (10 µg/mL) + MPP+(5 m*M*); Group VI: EGb761 (10 µg/mL) + MPP+(5 m*M*).	PC12 cells were pretreated for 24 h with E2, kaempferol, quercetin, EGb761 and Cp 202. Afterwards, MPP+ was added, and cells were incubated for 24 h.	-E2; -Kaempferol and quercetin; -EGb761 and CP 202.	The results suggest that both EGb761 and Cp 202 were able to rescue native and neuronal PC12 cells from MPP+-induced cell death.
[18] Cao *et al.* (2003)	Mice. Group I: Levodopa (50 mg/kg); Group II: Levodopa (50 mg/kg) + EGb (100 mg/kg).	Inject 6-OHDA in the mesencephalic ventral tegmental area and substantia nigra. Treated by Levodopa and Levodopa combined with EGb.	-Rotational behavioural observation; -TUNEL; -Immunocytochemistry; -Nissl’s body staining.	The combined use of EGb and levodopa may be feasible for the treatment of PD, being better than treatment with levodopa alone.
[19] Yang *et al.* (2001)	Wistar rats. Group I: saline solution (5 mL/kg, i.p.) + artificial fluid; Group II: saline solution + MPTP (20 µL/kg, brain area); Group III: EGb761 (50 mg/kg, i.p.) + MPTP; Group IV: EGb761 (100 mg/kg, i.p.) + MPTP.	The mice were pretreated with EGb for 19 days. One day later, MPTP was administered.	Contents of: -MDA; -SOD; -DA in substantia nigra.	EGb inhibited the decrease of DA and SOD and the increase of MDA. MPP+ induced PC12 cell apoptosis, and EGb prevented cellular apoptosis.
[20] Rojas *et al.* (2001)	Male C-57 black mice. Group I (n=6): saline solution + saline solution; Group II (n=6): EGb761 + saline solution; Group III (n=8): saline solution + MPP+; Group IV (n=8): EGb761 + MPP+. (EGb761 = 0.63, 1.25, 2.5, 5 or 10 mg/kg, i.p.; MPP+ = 1.18, 0.36 or 0.72 mg/kg)	The mice were pretreated with EGb761 for 17 days, followed by administration of MPP+.	-LP was analysed in the corpus striatum; -Striatal DA content was analysed by HPLC.	EGb761 (10 mg/kg) blocked MPP+ by 100%. Pretreatment with EGb761 partially prevented the dopamine-depleting effect of MPP+.
[21] Wu & Zhu (1999)	Female C-57 black mice. Group I: saline; Group II: MPTP; Group III: EGb761 (50 mg/kg, i.p.); Group IV: BN52020; Group V: BN52021; Group VI: MPTP + saline; Group VII: MPTP + EGb761 (50 mg/kg, i.p.); Group VIII: MPTP + saline; Group IX: MPTP + EGb761 (50 mg/kg, i.p.); Group X: EGb761 (20 mg/kg, i.p.) + MPTP; Group XI: EGb761 (50 mg/ kg, i.p.) + MPTP; Group XII: EGb761 (100 mg/kg, i.p.) + MPTP; Group XIII: BN52020 + MPTP; Group XIV: BN52020 + MPTP; Group XV: BN52021 + MPTP; Group XVI: BN52021 + MPTP. (MPTP = 30 mg/kg/d, i.p.; BN52020 or BN52021 = 10 or 50 mg/kg, i.p.; saline = 10 g/ml, i.p.)	Mice were randomly assigned to 16 groups. In the last seven groups, the animals were pretreated with EGb761, BN52020 or BN52021 for 7 days, then treated with the same extract 30 min before commencing with the MPTP injections for 6 days.	-DA levels; -MAO-B inhibition.	EGb761 treatment before or after MPTP administration effectively protects against MPTP, and the neuroprotective effect of EGb761 may, at least in part, be associated with its inhibitory effect on brain MAO.

Note: EGb = Ginkgo biloba extract; MPP+ = 1-methyl-4-phenyl-pyridine; MPTP = 1-methyl-4-phenyl-1,2,3,6-tetrahydropyridine ; DA = dopamine; TH = tyrosine hydroxylase; LP = lipid peroxidation; GPx = glutathione peroxidase; SOD = superoxide dismutase; 6-OHDA = 6-hydroxydopamine ; TBARS = thiobarbituric acid reactive substances; GSH = reduced glutathione; HPLC = high performance liquid chromatography; MAO = monoamine oxidase; E2 = 17β estradiol ; PD = Parkinson’s disease ; MDA = malondialdehyde; i.p. = intraperitoneal; icv = intracerebroventricular.

## ABBREVIATIONS

MPTP: = 1-methyl-4-phenyl-1,2,3,6- tetrahydropyridine
6-OHDA: = 6-hydroxydopamine
MPP^+^: = 1-methyl-4-phenyl pyridinium ion
EGb 761: = Extract of Ginkgo biloba 761
E_2_: = β-estradiol
DA: = dopamine
GPx: = glutathione peroxidase
GSH: = glutathione reductase
HPLC: = High-performance liquid chromatography
LP: = lipid peroxidation
MDA: = malondialdehyde
MAO: = monoamine-oxidase
PD: = Parkinson’s disease
SOD: = Superoxide dismutase
SNc: = *substantia nigra* pars compacta
TH: = tyrosine hydroxylase
VTA: = ventral tegmental area
